# Enhanced isopropanol–butanol–ethanol mixture production through manipulation of intracellular NAD(P)H level in the recombinant *Clostridium acetobutylicum* XY16

**DOI:** 10.1186/s13068-018-1024-0

**Published:** 2018-01-24

**Authors:** Chao Wang, Fengxue Xin, Xiangping Kong, Jie Zhao, Weiliang Dong, Wenming Zhang, Jiangfeng Ma, Hao Wu, Min Jiang

**Affiliations:** 1 State Key Laboratory of Materials-Oriented Chemical Engineering, College of Biotechnology and Pharmaceutical Engineering, Nanjing Tech University, Puzhu South Road 30#, Nanjing, 211816 People’s Republic of China; 2 Jiangsu National Synergetic Innovation Center for Advanced Materials (SICAM), Nanjing Tech University, Nanjing, 211816 People’s Republic of China

**Keywords:** *Clostridium acetobutylicum*, Isopropanol, NAD(P)H, pH control strategy, Calcium carbonate

## Abstract

**Background:**

The formation of by-products, mainly acetone in acetone–butanol–ethanol (ABE) fermentation, significantly affects the solvent yield and downstream separation process. In this study, we genetically engineered *Clostridium acetobutylicum* XY16 isolated by our lab to eliminate acetone production and altered ABE to isopropanol–butanol–ethanol (IBE). Meanwhile, process optimization under pH control strategies and supplementation of calcium carbonate were adopted to investigate the interaction between the reducing force of the metabolic networks and IBE production.

**Results:**

After successful introduction of secondary alcohol dehydrogenase into *C. acetobutylicum* XY16, the recombinant XY16 harboring pSADH could completely eliminate acetone production and convert it into isopropanol, indicating great potential for large-scale production of IBE mixtures. Especially, pH could significantly improve final solvent titer through regulation of NADH and NADPH levels in vivo. Under the optimal pH level of 4.8, the total IBE production was significantly increased from 3.88 to 16.09 g/L with final 9.97, 4.98 and 1.14 g/L of butanol, isopropanol, and ethanol. Meanwhile, NADH and NADPH levels were maintained at optimal levels for IBE formation compared to the control one without pH adjustment. Furthermore, calcium carbonate could play dual roles as both buffering agency and activator for NAD kinase (NADK), and supplementation of 10 g/L calcium carbonate could finally improve the IBE production to 17.77 g/L with 10.51, 6.02, and 1.24 g/L of butanol, isopropanol, and ethanol.

**Conclusion:**

The complete conversion of acetone into isopropanol in the recombinant *C. acetobutylicum* XY16 harboring pSADH could alter ABE to IBE. pH control strategies and supplementation of calcium carbonate were effective in obtaining high IBE titer with high isopropanol production. The analysis of redox cofactor perturbation indicates that the availability of NAD(P)H is the main driving force for the improvement of IBE production.

## Background

Biofuel generation from renewable resources has gained an increasing attention because of limited supply of fossil fuels and raising concerns on environmental issues. Biobutanol, naturally synthesized by solventogenic *Clostridia* through acetone–butanol–ethanol (ABE) fermentation process, has been considered as potential substitute for gasoline due to its more advanced fuel properties over bioethanol [1, 2]. The formative production of butanol with acetone and ethanol was even the second largest industrial fermentation process after ethanol production. However, the traditional ABE fermentation still encounters several big obstacles, such as high-product recovery costs caused by the low butanol concentration, and low butanol yield caused by the formation of by-products, such as acetone representing 30% in total mass of ABE, which result in economical un-competitiveness compared to the petrochemical process [3].

In ABE fermentation, acetone is considered as an undesirable product due to its poor fuel properties and corrosiveness to rubber parts of cars [4, 5]. Conversion of acetone into more value-added chemical or fuel offers a more promising strategy. In nature, several solventogenic *Clostridium* sp., such as *C. beijerinckii* NRRL B-593, have shown indigenous conversion of acetone into isopropanol, which possesses higher energy density than acetone (23.9 MJ/L vs 22.6 MJ/L) and shows broader usage as fuel, solvents, and chemical intermediates. In this biological process, the secondary alcohol dehydrogenase (s-ADH) encoding by *sadh* gene could efficiently catalyze acetone into isopropanol, resulting in an alcohol fuel mixture of isopropanol, butanol, and ethanol (IBE) [6–8]. Moreover, the fuel mixture in the fermentation broth has a direct end-usage as fuel additive, which would further eliminate the need for expensive recovery process and greatly improves the economic feasibility of IBE production.

In ABE fermentation, cofactors, such as NAD(H) and NADP(H), play essential roles in cell growth and synthesis of metabolic products. The intracellular level of NAD(P)H is a limited step in cofactor-dependent production systems, especially in the solventogenic *Clostridium* sp. [9, 10]. More NADPH will be required for IBE production, as s-ADH is NADPH-dependent enzyme. For example, after the introduction of s-ADH within solventogenic *Clostridum* sp., 4 and 2 mol NADH are required for generation of 1 mol butanol and ethanol, respectively. Meanwhile, 1 mol NADPH is also consumed for production of 1 mol isopropanol. The large amount requirement of reducing powers of NAD(P)H may increase the burden of bacterial cells and cause cofactor perturbation, especially in the recombinant strains. Inefficient regeneration of NAD(P)H caused by cofactor perturbation might further lower the cell growth and final solvent titer and yield. Hence, various strategies including supplementation of NAD(P)H precursors or process optimization et al. have been carried out to increase intracellular level of NAD(P)H. It is known that pH has been recognized as a key factor for ABE production through permission of the intracellular NADH level in solventogenic *Clostridium* sp. [11]. As solventogenic *Clostridium* sp. does not have the oxidative PP pathway, another route for NADPH generation is through the reaction of NAD kinase (NADK), which can catalyze the generation of NADP(H) from NAD(H) [12–14]. Research works have shown that NADK is a calcium-dependent enzyme, and it plays a key role in various biological activities of both prokaryotes and eukaryotes [15–17].

In the present study, *C. acetobutylicum* XY16 isolated by our lab was first genetically engineered to produce IBE mixtures through the simple introduction of s-ADH gene. Further redox analysis in parent and recombinant strains was carried out to detect the interaction between NAD(P)H and cell growth and IBE solvent production. Finally, process optimization using pH control strategies and supplementation of calcium carbonate were systematically investigated to increase cell growth and IBE production through regulation of intracellular level of NAD(P)H.

## Results

### IBE fermentation profile by the recombinant *C. acetobutylicum* XY16 harboring pSADH

**Fig. 1:**
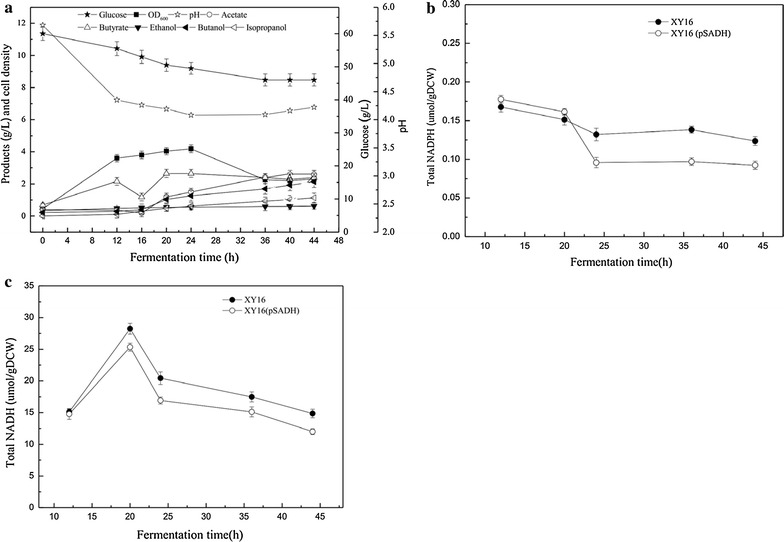
Fermentation profiles of *C. acetobutylicum* XY16 (pSADH) in batch fermentation mode under non-regulated pH strategy (**a**). Comparison of intracellular NADPH of *C. acetobutylicum* XY16 (pSADH) and *C. acetobutylicum* XY16 under non-regulated pH condition (**b**). Comparison of intracellular NADH of *C. acetobutylicum* XY16 (pSADH) and *C. acetobutylicum* XY16 under non-regulated pH condition (**c**)

After successful introduction of plasmid harboring pSADH into *C. acetobutylicum* XY16, batch fermentation without pH control was carried out to investigate the effects of *sadh* gene expression on solvent production and cell growth (Fig. 1). As expected, no residual acetone was detected in the culture medium after 44 h of fermentation. Instead, 1.13 g/L of isopropanol occurred after the expression of *sadh* in XY16 (pSADH) (Fig. 1a). During the fermentation process, pH values of the culture broth decreased from the initial value of 5.68 to around 4.23 in the acidogenesis phase, whereas the pH did not have a big fluctuation until the end of fermentation. It was observed that the maximum biomass reached 4.19 after 24 h of cultivation and then decreased rapidly. Further incubation did not increase the solvent production and glucose uptake with only 14.0 g/L of glucose consumed after 48 h of fermentation. The recombinant strain XY16 (pSADH) did not undergo a typical acid re-assimilation phase with the residual acetate and butyrate concentration reaching approximately 2.59 and 2.39 g/L, respectively. These results indicated that the metabolic profile of XY16 (pSADH) was dramatically affected by the expression of *sadh* gene. When entering the solventogenesis phase, cell growth began to decrease and IBE solvent production only reached 3.88 g/L, of which butanol, isopropanol, and ethanol concentrations were 2.13, 1.13, and 0.62 g/L, respectively.

To better understand the discrepancy shifting from ABE to IBE, the total levels of NADPH and NADH were further examined (Fig. 1b). As seen, when solvent production was initiated during 20–25 h, the NADPH levels of parent strain XY16 were kept at a stable level of about 0.15 μmol/g DCW, while those of the recombinant XY16 (pSADH) were dramatically decreased from the initial level of 0.17 μmol/g DCW to a lower level of 0.07 μmol/g DCW, indicating that the synthesis of isopropanol enlarged the demand of NADPH (Fig. 1b). This is also consistent with our expectation that more isopropanol synthesized, more NADPH consumed. On the other hand, the NADH levels showed a typical characteristic that continuously increased through the acidogenesis phase and decreased in the solventogenesis phase (Fig. 1c). However, the NADH level of XY16 (pSADH) was lower than that of XY16 and the total amount of NADPH and NADP^+^ within XY16 (pSADH) was increased (data not shown), suggesting that NADH was phosphorylated by NADK to support cell growth and solvent production. It is known that the reducing agent, NADPH, is an important coenzyme required in several biological reactions, especially for cell growth [7, 9]. The further driving force for isopropanol synthesis increased the demand of NADPH, resulting in low level of NADPH and NADH. To increase the solvent flux, it is crucial to provide constant NADH and NADPH to achieve a balanced redox status for cell growth and IBE production.

### High IBE production by the recombinant *C. acetobutylicum* XY16 (pSADH) through pH regulation

**Fig. 2:**
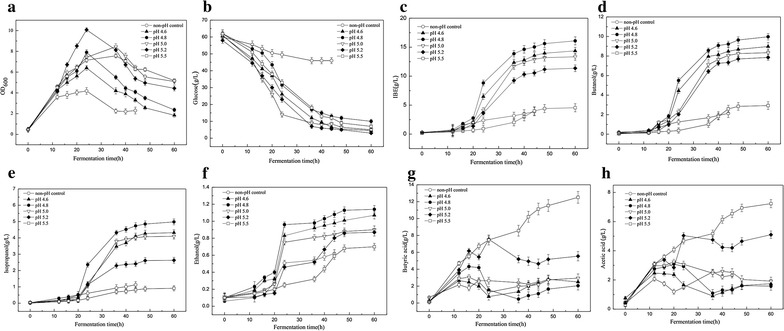
Batch fermentation profiles of *C. acetobutylicum* XY16 (pSADH) under different pH conditions in 5 L bioreactors. **a** Profiles of cell growth (OD_600_) in batch fermentations and pH controlled at 4.6, 4.8, 5.0, 5.2, and 5.5. **b** Profiles of glucose consumption in batch fermentations and pH controlled at 4.6, 4.8, 5.0, 5.2, and 5.5. **c** Profiles of IBE concentration in batch fermentations and pH controlled at 4.6, 4.8, 5.0, 5.2, and 5.5. **d** Profiles of butanol concentration in batch fermentations pH controlled at 4.6, 4.8, 5.0, 5.2, and 5.5. **e** Profiles of isopropanol concentration in batch fermentations and pH controlled at 4.6, 4.8, 5.0, 5.2, and 5.5. **f** Profiles of ethanol concentration in batch fermentations and pH controlled at 4.6, 4.8, 5.0, 5.2, and 5.5. **g** Profiles of butyric acid concentration in batch fermentations and pH controlled at 4.6, 4.8, 5.0, 5.2, and 5.5. **h** Profiles of acetic acid concentration in batch fermentations and pH controlled at 4.6, 4.8, 5.0, 5.2, and 5.5

Cofactor manipulation could potentially be a powerful and economical strategy for the improvement of overall IBE production [9]. pH has been reported as a critical factor for ABE fermentation, which greatly influences the production of solvents through regulation of the intracellular level of NAD(P)H. However, the optimal pH varied depending on the culture conditions and strains [1, 18]. Thus, the effect of various pH values on IBE production by the recombinant *C. acetobutylicum* XY16 (pSADH) was further investigated. Batch fermentations were performed at pH values of 4.6, 4.8, 5.0, 5.2, and 5.5 in the modified P2 medium. As shown in Fig. 2, cell growth and IBE production were significantly affected by pH values. Under these conditions, the growth of engineered strain recovered to that of the parent strain XY16 and showed the typical biphasic fermentation profile of *C. acetobutylicum* (Fig. 2a) [1]. When pH was maintained at low value of 4.6, the maximum cell density of 6.40 (OD_600_) was obtained at 24 h (Fig. 2a) and the final IBE concentration reached 14.36 g/L (Fig. 2c). However, when pH was maintained at 5.2 or 5.5, a decrease in IBE production was observed, and the metabolic flux shifted towards acids production rather than solvents, resulting in high acids production with 12.51 g/L of butyric acid and 7.23 g/L of acetic acid (Fig. 2g, h). Similar results have been reported in other studies, in which more acids were produced at higher pH values than lower ones [19–21]. When pH was controlled at 4.8, the maximal IBE production of 16.09 g/L was obtained, of which the concentration of butanol, isopropanol, and ethanol was 9.97, 4.98, and 1.14 g/L, respectively (Fig. 2d–f). Meanwhile, the recombinant strain XY16 (pSADH) utilized glucose more efficiently at pH 4.8 with a high glucose consumption rate of 0.86 g/L/h (Fig. 2b). It can be concluded that maintenance of optimal pH at 4.8 for recombinant strain XY16 (pSADH) not only favored cell growth during acidogenesis, but also improved IBE production during solventogenesis.

### Detection of NADH and NADPH levels in the recombinant *C. acetobutylicum* XY16 (pSADH)

**Fig. 3:**
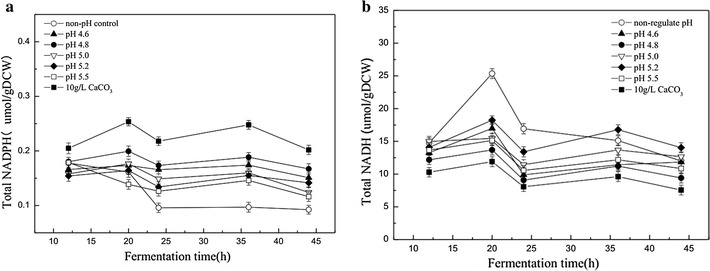
Effects of intracellular NADPH at different fermentation time and pH controlled at 4.6, 4.8, 5.0, 5.2, and 5.5 (**a**). Effects of intracellular NADH at different fermentation time and pH controlled at 4.6, 4.8, 5.0, 5.2, and 5.5 (**b**)

The previous studies have shown that the introduction of *sadh* cannot completely convert acetone to isopropanol in some solventogenic *Clostridium* sp. For example, although the transformant *C. acetobutylicum* ATCC 824 (pFC007) after overexpression of ctfA/B genes along with *sadh* showed high capacity for conversion of acetone into isopropanol (> 95%), however, the residual acetone of 0.9 g/L was still detected [8]. Different from those studies, acetone could be completely converted into isopropanol by the recombinant strain XY16 (pSADH), but the total IBE production was only 3.88 g/L without pH control. Only through pH control strategy, the maximum IBE production was increased up to 16.09 g/L. To elaborate the underlying mechanisms, the intracellular levels of NADPH and NADH were investigated. As shown in Fig. 3, during acidogenesis, the intracellular NADH increased rapidly with cell growth (Figs. 2a, 3b). NADH has been reported as one of the major contributing factors for solvent production [1]. Supplementation of NADH precursors could efficiently improve solvent production. For example, addition of nicotinamide (VB3) could obviously improve both s-ADH and butanol dehydrogenase (BDH) activities and improve solvent production [22]. As shown in Fig. 3b, after 20 h of fermentation, NADH as the main reducing power was consumed to synthesize the solvents and regeneration of NAD^+^. Thus, plenty of NADH was created by consuming large amount of glucose, accompanied with a dramatic increase in the cell biomass and solvent production (Fig. 2b, c). Compared to other pH conditions, the NADH level at pH 4.8 did not increase obviously, due to the fact that the produced NADH was mostly consumed for the solvent production. Levels of NADPH in the pH-controlled fermentation were much higher than those in the extract from the control (Fig. 1b). In addition, the increase of NADPH during solventogenesis phase indicated that more reducing equivalents were produced in the forms of NADPH. This trend also clearly indicated by the increase of isopropanol concentration. Therefore, the increased solvent production was attributed to the improved availability of intracellular NADH and NADPH.

### High IBE production by the recombinant *C. acetobutylicum* XY16 (pSADH) through supplementation of calcium carbonate

**Fig. 4:**
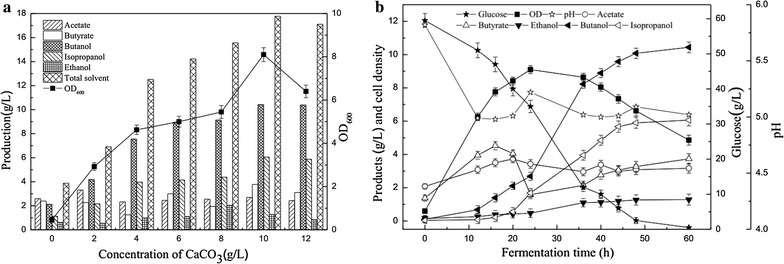
Fermentation results of *C. acetobutylicum* XY16 (pSADH) under different CaCO_3_ concentrations. Cells were grown in serum bottles with 60 g/L glucose for 72 h (**a**). The profiles of *C. acetobutylicum* XY16 (pSADH) in batch fermentation under 10 g/L CaCO_3_ (**b**)

NADPH can be generated from NADP^+^ and NADK is the sole enzyme catalyzing the generation of NADP^+^ from NAD^+^ [23]. It has been reported that NADK can be activated by the factors of calcium ions [24, 25]. Hence, CaCO_3_ was added into the fermentation medium, which may play dual roles for pH adjustment and activation of NADK. Accordingly, various amounts of calcium carbonate (0, 2, 4, 6, 8, 10, and 12 g/L) were supplemented into the medium and batch fermentations were carried out for 72 h using the recombinant *C. acetobutylicum* XY16 (pSADH) (Fig. 4a). In the control batch without CaCO_3_, the biomass (OD_600_) reached 0.47 and only 2.13 g/L of butanol was produced when cultured in anaerobic bottles. When the dosage of calcium carbonate increased to 2 and 4 g/L, the butanol production increased to 4.19 and 7.56 g/L, respectively. As shown in Fig. 4a, a small quantity of CaCO_3_ led to the rapid cell growth of strain XY16 (pSADH). Meanwhile, the glucose utilization by strain XY16 (pSADH) was also increased in P2 medium supplemented with CaCO_3_. Both cell growth and final IBE concentration increased along with the increase of CaCO_3_ concentration. The highest IBE production of 17.77 g/L and cell density of 8.10 were achieved in a serum bottle medium spiked with 10 g/L CaCO_3_. In addition, no significant difference in glucose utilization and IBE production was observed when XY16 (pSADH) was cultivated in P2 medium containing above 10 g/L CaCO_3_.

To further investigate solvent production profiles by strain XY16 (pSADH) under optimal CaCO_3_ concentration, batch fermentation in 5 L fermentor was carried out (Fig. 4b). When 10 g/L of calcium carbonate was supplemented, the initial pH value of the fermentation medium was 5.82. With the increase of fermentation duration, the pH value decreased to around 4.9, which is the optimal pH for IBE fermentation (Fig. 2). In addition, it is worth noting that the IBE concentration increased dramatically from 3.07 to 17.77 g/L, with a glucose consumption rate of 0.99 g/L/h. Cell growth was also significantly improved in the presence of CaCO_3_, and the biomass of XY16 (pSADH) increased up to 9.10. Hence, supplementation of CaCO_3_ provides a favorable pH range for the growth of XY16 (pSADH), which also contributed to the increase of IBE production.

In addition to acting as the buffering agent, CaCO_3_ could also increase the NADPH availabilities (Fig. 3). Compared with pH control strategy, the level of NADPH was increased to the highest (Fig. 3a). When CaCO_3_ was added, the driving force from Ca^2+^ could enhance the reaction from NAD(H) to NADP(H), and consequently, the isopropanol concentration reached 6.06, which was 22% higher than that at pH 4.8. It is also worth noting that total solvent production was increased by 10% from 16.09 to 17.77 g/L with 10.51, 6.02, and 1.24 g/L of butanol, isopropanol, and ethanol, respectively (Fig. 4). Meanwhile, high solvent yield of 0.312 g/g was obtained compared with at pH 4.8. In our current study, it suggests that improving the availability of NAD(P)H is an efficient approach for increasing IBE solvent production.

## Discussion

In nature, *C. beijerinckii* strains, mainly *C. beijerinckii* NRRL B-593, were shown to indigenously produce isopropanol without acetone formation, but ranges of isopropanol titer still maintained at low levels and are inconclusive based on different studies (Table 1). According to Survase et al, 2.16 and 3.71 g/L of isopropanol and butanol from 60 g/L of glucose were produced by *C. beijerinckii* NRRL B-593; however, ethanol production was not reported [26]. Shaheen et al. demonstrated that *C. beijerinckii* NRRL B-592 produced approximately 16 g/L of total solvents including isopropanol from 80 g/L of maize mash, but the efficiency of acetone conversion is still unknown [27]. Ng et al. found that IBE were all produced by *C. beijerinckii* NRRL B-592 from 60 g/L of glucose with a final titer of 2.22, 1.85, and 8.13 g/L, respectively [28]. More recently, Xin et al. isolated another native isopropanol producer, *Clostridium* sp. strain NJP7. With enhancement of buffering capacity and alcohol dehydrogenase activities through supplementation of NADPH precursor—VB3, butanol and isopropanol titer were finally improved to 12.21 and 1.92 g/L, respectively [22].

**Table 1:** Comparison of IBE production using wild-type or genetically modified solventogenic *Clostridium* species

Bacterium	Substrate	Metabolic products (g/L)	Solvent yield (g/g)	Ref.
*C. beijerinckii* NRRL B-593	Glucose 60 g/L	Isopropanol (2.16) Butanol (3.71)	0.21	[26]
*C. beijerinckii* NRRL B-593	Glucose 60 g/L	Isopropanol (1.85) Butanol (8.13) Ethanol (2.22)	0.33	[28]
*Clostridium* sp. NJP7	Glucose 60 g/L	Acetone (2.21) Isopropanol (1.92) Butanol (12.21)	0.24	[22]
*C. acetobutylicum* 824 PJC4BK (pIPA3-Cm2)	Glucose 132.9 g/L	Isopropanol (4.4) Butanol (14.1) Ethanol (1.9)	0.2	[4]
*C. acetobutylicum* 824 BKM19 (pIPA100)	Glucose 76 g/L	Isopropanol (3.5) Butanol (15.4) Ethanol (9.6)	0.38	[29]
*C. acetobutylicum* 824 *Δbuk pCLF952*	Glucose 63.5 g/L	Acetone (0.36) Isopropanol (4.75) Butanol (14.63) Ethanol (1.01)	0.33	[30]
*C. acetobutylicum* XY6 (pSADH)	Glucose 60 g/L	Isopropanol (6.02) Butanol (10.51) Ethanol (1.24)	0.31	This study

Different from native isopropanol producers, the recombinant strains via introduction of *sADH* show much stable solvent production levels and could efficiently switch ABE into IBE; however, the isopropanol titer varied widely depending on the strains and culture conditions. For example, through overexpression of a synthetic acetone operon (*adc*, *ctfA*, *ctfB*) and sADH in a *buk* gene deletion mutant of *C. acetobutylicum* ATCC 824, the final titers of isopropanol, butanol, and ethanol were improved to 4.4, 14.1, and 1.9 g/L, respectively [4]. The expression of *sADH* in a hyper ABE producing BKM19 strain gave 28.5 g/L of IBE with 3.5, 15.4, and 9.6 g/L of isopropanol, butanol, and ethanol from 76.0 g/L glucose with negligible amount of acetone (0.4 g/L) in the large-scale (200 L) batch fermentation [29]. Similarly, when Dusséaux et al. constructed the isopropanol synthetic pathway in a butyrate minus mutant strain (*C. acetobutylicum* ATCC 824 Δbuk pCLF952), the final biofuel titer of 20.75 g/L with a yield of 0.33 g/g glucose was obtained, and the acetone, isopropanol, butanol, and ethanol titer reached 0.36, 4.75, 14.63, and 1.01 g/L [30]. In the current study, the recombinant *C. acetobutylicum* XY16 harboring pSADH was also successfully constructed, adding to the pool of IBE butanol generating microbes. Different from those native or genetically modified isopropanol producers, strain XY16 (pSADH) could generate much higher isopropanol production of 6.02 g/L through regulation of NAD(P)H levels. Similar amount of IBE (17.77 g/L) was obtained with yield and productivity of 0.312 g/g and 0.30 g/L/h (Table 1). Further studies to improve final butanol titer and tolerance are still needed through chemical random mutagenesis or adaptive evolution.

Cofactor manipulation has been proven as a powerful tool for improvement of overall process yield and productivity in ABE fermentation process. To increase the IBE flux, it is crucial to provide constant NADH and NADPH to achieve a redox balance for enhanced IBE production, as the BDH and s-ADH are NADH- and NADPH-dependent, respectively [20, 22]. A direct and simple method to achieve this goal is to supplement some precursors of NADH and NADPH, such as nicotinic acid (NA), VB3, etc. For example, NADPH-dependent s-ADH activities within the native isopropanol producer of *Clostridium* sp. NJP7 were 1.5 times higher in the presence of 20 mg/L of VB3, leading to the increase of isopropanol production from 0.55 to 0.72 g/L. Meanwhile, 1.6 times increase of BDH was observed in the presence of VB3 than that in the control and the final butanol production of 6.28 g/L occurred in the medium broth [22]. Similarly, the increased IBE production from 3.88 to 16.09 g/L by the recombinant strain XY6 (pSADH) was also observed only through pH regulation, which may be attributed to the improved availability of intracellular NADH and NADPH (Figs. 2c, 3). However, it should be noticed that supplementation of additional precursors would add on the economics of the whole IBE fermentation process. Alternatively, this pH control strategy offers a promising way. Interestingly, addition of cheap CaCO_3_ could further enhance final IBE production to 17.77 g/L, which may play dual roles as both buffering agency and activators of NADPH (Fig. 4). pH could be maintained at optimal levels when supplemented with CaCO_3_, and further proteomic analysis has also shown that calcium could not only elevated bacterial growth and sugar utilization, but also improved butanol tolerance and direct enhanced activities of key solventogenic enzymes in solventogenic *Clostridium* sp. [31, 32]. When using strain XY6 (pSADH), it was further found that the level of NADPH was increased to the highest levels in the presence of calcium, leading to the highest isopropanol production of 6.06 g/L (Table 1). Calcium has been reported as an activator for NAD kinase (NADK). Hence, supplementation of calcium could further facilitate IBE production through regulation of NADH and NADPH levels [31]. However, further specific analyses of complex global response to calcium within the recombinant strain XY6 (pSADH) are required to comprehensively investigate the mechanisms on improvement of IBE production.

## Conclusion

*Clostridium acetobutylicum* XY16 (pSADH) was successfully metabolically constructed to produce IBE fuel mixtures with complete elimination of acetone. The analysis of redox cofactor perturbation indicated that the availability of NAD(P)H was the main target to improve IBE production. Both pH control strategy and calcium carbonate could increase the intracellular NAD(P)H levels and IBE concentration. Under the optimal pH level of 4.8, the total IBE production was significantly increased from 3.88 to 16.09 g/L. Especially, the addition of 10 g/L of calcium carbonate could further increase the IBE production to 17.77 g/L. Based on this, it can be concluded that improvement of the availability of NAD(P)H is an efficient approach for enhancement of IBE production.

## Methods

### Strains and cultivation conditions

*Clostridiumacetobutylicum* XY16 was screened by our laboratory and stored in China Center for Type Culture Collection (CCTCC No. M 2010011) [33]. All *C. acetobutylicum* strains were routinely cultured at 37 °C in an anaerobic chamber (Bug Box, Ruskinn Technology, Leeds, UK) filled with 80% N_2_, 10% CO_2_, and 10% H_2_.

Yeast extract/peptone/starch (YPS) medium was used as culture medium containing (per liter) 3.0 g yeast extract, 5.0 g peptone, 10.0 g soluble starch, 2.0 g CH_3_COONH_4_, 2.0 g NaCl, 3.0 g MgSO_4_·7H_2_O, 1.0 g KH_2_PO_4_, 1.0 g K_2_HPO_4_, and 0.1 g FeSO_4_·7H_2_O. The initial pH was adjusted to pH 6.0 with 1 M HCl. Erythromycin was added at concentration of 20 mg/L if necessary.

### Plasmid construction and transformation

The fragment of *sadh* gene was synthesized according to the sequence in NCBI with accession number AF157307, using primers sADH-1: 5′-CGCGGATCC ATGAAAGGTTTTGCAATGCTAGGTATTTAATAAGTT-3′and sADH-2: 5′-CCGG AATTCTTATAATATAACTACTGCTTTAATTAAGTC-3′. The resulted fragment was then ligated into the *E. coli*/*Clostridium* shuttle vector pIMP1 under its nature promoter of *ptb* [34, 35]. Plasmid DNA from *E. coli* strains was extracted using the Axygen plasmid miniprep kit.

Plasmid DNA was introduced into competent *E. coli* TOP 10 harboring plasmid pAN2 for methylation prior to transformation into *C. acetobutylicum* as described earlier. Methylated pIMP1-*ptb*-*sadh* was then electroporated into *C. acetobutylicum* XY16 as described by Mermelstein [36–38]. The transformants were screened using PCR amplification of the *sadh* gene. The cells were transferred and grown on agar plates containing erythromycin and then cultivated in YPS liquid medium.

### Fermentation conditions

Throughout these studies, a 10% (v/v) actively growing cell suspension was inoculated, and nitrogen gas was purged to remove oxygen. *C. acetobutylicum* strains were grown in modified P2 medium (per liter): KH_2_PO_4_, 0.5 g; K_2_HPO_4_, 0.5 g; CH_3_COONH_4_, 2.2 g, MgSO_4_·7H_2_O, 0.2 g; MnSO_4_·H_2_O, 0.01 g; FeSO_4_·7H_2_O, 0.01 g; NaCl, 0.01 g; corn steep liquor, 1 g [39]. When necessary, erythromycin (20 mg/L) was added prior to inoculation.

The pH-controlled batch fermentations were performed in a 5 L fermentor (Bioflo 110, USA). The initial fermentation broth (2 L) was sterilized at 121 °C for 15 min. The glucose solution was sterilized separately and added to culture medium to give a final concentration of 60 g/L.

Nitrogen was purged into the medium to remove oxygen before and after inoculation, and the temperature was maintained at 37 °C with agitation at 120 rpm. The pH was maintained at different level with automatic addition of 2 M HCl and 2 M NaOH. To determine the optimal CaCO_3_ concentration for IBE production, fermentations were conducted for 72 h supplemented with 2, 4, 6, 8, 10, or 12 g/L of CaCO_3_. CaCO_3_ was sterilized by dry heat sterilization at 160 °C for 30 min before being added into the medium. Precultures grown in YPS medium (10%) were transferred into loosely capped 100-mL Pyrex medium bottles containing 40 mL P2 medium. P2 medium without CaCO_3_ was used as the control. Unless otherwise stated, all fermentations were conducted in triplicate, and averages of parameters were reported.

### Analytical methods

OD_600_ was analyzed using an ultraviolet–visible spectrophotometer (Spectrumlab 752S). Dry cell weight (DCW) was calculated as follows: DCW (g/L) = 0.26 × OD_600_. Glucose was analyzed using an SBA-40C biosensor analyzer (Institute of Biology, Shan-dong Province Academy of Sciences, PR China). Acetate, butyrate, ethanol, isopropanol, acetone, and butanol concentrations were measured in duplicate using high-performance liquid chromatography (HPLC) analysis (Chromeleon server monitor, P680 pump, Dionex, USA) equipped with UV and refractive index (RI) detectors. The supernatant was filtered by a 0.2-μm nylon filter before being injecting to HPLC. An aminex HPX-87H organic acid analysis column (7.8 × 300 mm) (Bio-Rad Laboratories, Inc., CA) was maintained at 15 °C with 0.05 mM H_2_SO_4_ as mobile phase and at a flow rate of 0.5 mL/min. Total solvent was the sum of isopropanol (acetone), butanol, and ethanol. The solvent yield is defined as the amount of solvent produced from 1 g of totally consumed sugar (expressed in g/g).

### NAD^+^/NADH and NADP^+^/NADPH assay

Intracellular concentrations of NAD(P)H and NAD(P)^+^ were assayed using the cycling method [22, 40]. Briefly, cells were harvested by centrifugation at 12,000 rpm and 4 °C. After that, 300 μL of 0.2 M NaOH (for NAD(P)H extraction) or 300 μL 0.2 M HCl (for NAD(P)^+^ extraction) was added to re-suspend the pellets. The cell lysates were kept at 50 °C for 10 min and then cooled to 0 °C and centrifuged. The extracts were neutralized by adding 300 μL of 0.1 M HCl (for NAD(P)H extraction) or 300 μL of 0.1 M NaOH (for NAD(P)^+^ extraction) dropwise while vertexing. The cellular debris was removed by centrifuging at 12,000 rpm for 10 min at 4 °C. For the measurement of intracellular NAD^+^/NADH or NADP^+^/NADPH levels, 50 μL of neutralized extract, 300 of μL ddH_2_O, and 600 μL of assay mixture containing 1.0 M of *N*,*N*-bis(2-hydroxyethyl)glycine (bicine) buffer (pH = 8.0), ethanol/glucose 6-phosphate, 40 mM of EDTA (pH = 8.0), 4.2 mM of 3-(4,5-dimethyl-2-thiazolyl)-2,5-diphenyl-2-*H*-tetrazolium bromide (MTT), and 16.6 mM of phenothiazine methyl sulfate (PMS) were added to 1 mL cuvettes. The reaction was initiated by adding 50 μL of alcohol dehydrogenase [500 units/mL, for NAD(H)] or glucose 6-phosphate dehydrogenase (70 units/mL, for NAD(P)H). The absorbance at 570 nm was determined. All experiments were performed in triplicate.

## Abbreviations

ABE: acetone–butanol–ethanol
IBE: isopropanol–butanol–ethanol
s-ADH: secondary alcohol dehydrogenase
NADK: NAD kinase
BDH: butanol dehydrogenase
VB3: nicotinamide
